# Serotyping and Antimicrobial Susceptibility Pattern of* Escherichia coli* Isolates from Urinary Tract Infections in Pediatric Population in a Tertiary Care Hospital

**DOI:** 10.1155/2016/2548517

**Published:** 2016-03-07

**Authors:** Shweta Sharma, Nirmaljit Kaur, Shalini Malhotra, Preeti Madan, Wasim Ahmad, Charoo Hans

**Affiliations:** Department of Microbiology, PGIMER and Dr. RML Hospital, New Delhi 110001, India

## Abstract

Urinary tract infections (UTIs) in pediatric population are associated with high morbidity and long term complications. In recent years, there is increased prevalence of* Escherichia coli *(*E. coli*) strains producing extended spectrum *β*-lactamase, Amp C, and Metallo *β*-lactamase, making the clinical management even more difficult. This study was aimed to detect the serotypes and to determine antimicrobial susceptibility profile of* E. coli *isolates from urine samples of children <10 yrs old. A total of 75 pure* E. coli *strains isolated from patients with symptoms of UTI and colony count ≥10^5^ organisms/mL were included in the study. Antibiotic sensitivity pattern showed maximum resistance to nalidixic acid (98.7%), followed by ampicillin (97.3%), amoxi-clavulanate (96%), and fluoroquinolones (92%) while most of the isolates were found sensitive to piperacillin-tazobactam (13.3%), nitrofurantoin (5.3%), and meropenem (1.3%). 48% of the strains were ESBL producer (extended spectrum beta lactamase). 44% strains were typable withantisera used in our study and the most common serogroup was O6 (33.3%) followed by O1 (15.1%) and O15 (15.1%). To conclude, judicious use of antibiotics according to hospital antibiotic policy and infection control measures should be implemented to prevent spread of multidrug resistant organisms.

## 1. Introduction

Urinary tract infections (UTIs) are one of the most common infections encountered in the clinical practice, and in pediatric population it is associated with high morbidity and long term complications like renal scarring, hypertension, and chronic renal failure [1]. Early diagnosis, proper investigation, adequate therapy, and prolonged careful follow-up in children with UTI will decrease chronic renal failure in adults. UTI is mainly associated with members of the* Enterobacteriaceae* family and* Escherichia coli* (*E. coli*) are the most predominant pathogen causing UTIs [2].* E. coli* that are associated with UTI are commonly named uropathogenic isolates, although there is evidence that different pathotypes may be related to UTI; however, uropathogenic* E. coli* (UPEC) is responsible for approximately 90% of all UTIs [3]. Uropathogenic* E. coli* (UPEC) possess virulence factors which help them to colonize the periurethral area, enter urinary tract, and cause retrograde infection.* E. coli* strains are normally identified by serological typing of their H (flagellar), O (lipopolysaccharide), and, in some cases, K (capsular) surface antigens. Since, 176 O-serogroups had been described for* E. coli* [3]. In UPEC, the O-serogroups are related to the virulence factor profile of each strain. Previous studies reported that O1, O2, O4, O6, O7, O8, O15, O16, O18, O21, O22, O25, O75, and O83 serogroups are preferentially associated with UPEC strains [4, 5]. Each serotype of* E. coli* has an important role in clinical presentation of UTI, and the prevalence of different serotypes varies in different regions. In recent years, there has been an increase in the resistance to cephalosporins, fluoroquinolones, and trimethoprim among UTI cases, which is a growing cause of concern. In hospitalized patients, there is increased prevalence of* E. coli* strains producing extended spectrum *β*-lactamase, Amp C, and Metallo-*β*-lactamase, making the clinical management even more difficult [6, 7]. Also clinicians must be aware of the susceptibility patterns of UPEC strains in their specific geographical populations to optimize the use of empirical antibiotic therapy for UTIs. This study was aimed to detect the serotypes and to determine antimicrobial susceptibility profile of* E. coli* isolates from urine samples of children less than 10 yrs old.

## 2. Materials and Methods

This cross-sectional observational study was conducted in the Department of Microbiology in a tertiary care centre, New Delhi. The samples were collected from children <10 yrs admitted in pediatric ward, ICU (intensive care unit), and also from children whoattended OPD (out-patient department) with symptoms of UTI. Informed consent was taken from all patients included in the study. Urine specimen was obtained through catheterization, suprapubic aspiration (SPA), or midstream urine after thorough cleaning of the perineal region [8, 9]. The freshly collected urine samples were inoculated on 5% sheep blood agar and MacConkey (MCA) agar and then incubated at 37°C for 24 hrs. Culture plates with a growth of single morphotype of* E. coli* having colony counts ≥105 CFU/mL were considered significant. A total of 75* E. coli* strains isolated over a period of one year from January 2014 to December 2014 were included in the study.* E. coli* isolates were identified on the basis of colony morphology and various biochemicals like motility, oxidase, sugars, indole, methyl red, Voges Proskauer, citrate, urease, triple sugar iron agar, lysine and ornithine decarboxylase and arginine dihydrolase, and so forth [10]. Also the isolates were confirmed in automated system, that is, Microscan WalkAway 40 plus system. All the isolates were tested for antimicrobial susceptibility by Kirby Bauer's disc diffusion method and zone diameters were interpreted according to the Clinical and Laboratory Standards Institute (CLSI) guidelines [11]. The antibiotics tested were ampicillin (10 *μ*g), gentamicin (10 *μ*g), amikacin (30 *μ*g), nalidixic acid (30 *μ*g), nitrofurantoin (300 *μ*g), cefotaxime (30 *μ*g), ceftazidime (30 *μ*g), amoxy-clavulanic acid (20/10 *μ*g), piperacillin-tazobactam (100/10 *μ*g), meropenem (10 *μ*g), and cotrimoxazole (1.25/23.75 *μ*g) (Hi-Media Pvt. Laboratories, Bombay, India). Quality control strain* E. coli* ATCC 25922 was used to validate the results of the antimicrobial discs. ESBL production was determined by using phenotypic confirmatory test: antibiotic disc of ceftazidime 30 *μ*g and ceftazidime-clavulanic acid 30/10 *μ*g and also cefotaxime 30 *μ*g and cefotaxime-clavulanic acid 30/10 *μ*g (Hi-Media) were placed on inoculated Mueller-Hinton Agar (MHA) media 30 mm apart from centre to centre and incubated at 37°C for 16–18 h. The zone of inhibition was recorded and difference in zone size of 5 mm or more for either antimicrobial agents tested in combination with clavulanic acid versus its zone when tested alone were considered ESBL [11]. Serotyping of* E. coli* strains was performed using 7 different monoclonal* E. coli* (EPEC) test sera (Statens Serum Institute) according to manufacturer's recommendations. Polyvalent antisera used in our study included O1, O2, O4, O6, O7, O15, and O75 serotypes. The isolate giving positive reaction with polyvalent antisera was retested with individual antisera. Colony of* E. coli* was inoculated in Brain Heart Infusion (BHI) broth and incubated overnight at 37°C. After boiling for 1 hr, 80 *μ*L of supernatant was mixed with equal quantity of antiserum in a microtitre plate and incubated overnight. Carpet formation was taken as positive reaction and button formation as negative reaction [12]. All the data were entered in the Microsoft excel sheet and processed for statistical analysis to calculate various percentages.

## 3. Results

Out of seventy-five* E. coli* strains isolated from children, male : female ratio was found to be 1.2 : 1 with 54.7% of the patients being males. 66.7% of the isolates were derived from admitted patients while 33.3% of the patients came to OPD with symptoms of UTI. 54.7% of the isolates were from ward patients followed up by OPD (33.3%) and ICU (12%) (Table 1). Admitted patients developed UTI after admission in the hospital and were having hospital acquired UTI with risk factors like catheterization (72%), antibiotic use (96%), and immunosuppression (22%). OPD patients came with symptoms of UTI and were labelled as community acquired UTI with no risk factors. Antibiotic sensitivity pattern of uropathogenic* E. coli* showed maximum resistance to nalidixic acid (98.7%), followed by ampicillin (97.3%), amoxi-clavulanate (96%), and trimethoprim-sulfamethoxazole (84%) while most of the isolates were found sensitive to piperacillin-tazobactam (13.3%), nitrofurantoin (5.3%), and meropenem (1.3%). 36 (48%) of the strains were ESBL producer (extended spectrum beta lactamase) (Table 2). Majority of the isolates (90%) were multidrug resistant (≥3 classes of antimicrobials) and one strain was found resistant to carbapenem group, while none of the strains were sensitive to all the antibiotics tested (Table 2). A total of 33 (44%) strains were typable with antisera used in our study while 42 (56%) were nontypable. The most common serogroup was O6 (33.3%) followed by O1 (15.1%) and O15 (15.1%) as shown in Table 3. The antimicrobial susceptibility pattern was similar among typable strains and nontypable strains as shown in Table 2.

## 4. Discussion

Our study describes the distribution, antibiotic susceptibility pattern, and serogroups of* E. coli* isolated from children < 10 yrs with symptoms of UTI and colony count more than 10^5^ organisms/mL in our hospital. There was an overall male preponderance (54.7%) which is in accordance with other studies [1, 13]; however other studies suggest higher prevalence of UTI among females compared to males [14, 15]. During the first year of life, boys have a higher incidence of UTI of about 2.7% compared to girls (0.7%) while in all other age groups, girls are more prone to developing UTI. In our study, males were more commonly involved till 5 yrs of age, while females are more common between 5 and 10 yrs of age [9].* E. coli* have several factors responsible for their attachment to the uroepithelium like adhesin, pili, fimbriae, and P1-blood group phenotype receptor [16]. Antibiotic resistance has increased over years, varies from country to country, and is a major clinical problem in treating infections caused by these microorganisms. Maximum isolates (85–90%) showed high resistance to cefotaxime, norfloxacin, nalidixic acid, cotrimoxazole, and amoxy-clavulanic acid while resistance to gentamicin and amikacin was found to be 46.7% and 16%, respectively. Amikacin has to be administered parenterally and it is nephrotoxic. However, isolates were found more sensitive to nitrofurantoin (5.3%) and piperacillin-tazobactam (13.3%). These findings were in accordance with other studies [17, 18]. Increased resistance might be due to widespread, inappropriate use of antibiotics and production of extended spectrum beta lactamases in these isolates [18, 19]. In our study, 48% of* E. coli* strains were found to produce ESBL which is consistent with other Indian studies [17, 20]. Carbapenems are the drug of choice for isolates producing ESBL as carbapenem group is highly stable against *β*-lactamase. In the present study, all the isolates except one were found to be sensitive to carbapenems (meropenem). With increasing resistance among most antibiotics, a urine culture with sensitivity pattern of isolates should be obtained before starting treatment.

Only limited O-serogroups of* E. coli* like O1, O2, O4, O6, O7, O15, O21, O22, O25, O75, O83, and so forth have been associated with UTI and their prevalence varies from place to place and time to time. In our study, 44% of* E. coli* isolates were found to react with polyvalent O-serogroups and the most common O-serogroup was O6 (33.3%), followed by O1 (15.1%), O15 (15.1%), and O75 (12.1%). Similarly, O6 was the most common serotype isolated in* E. coli* causing UTI in other studies [12, 21]. However, few studies suggest O1 as the most common serotype among UTI isolates, which is the second most common serotype among typable strains in our study [22]. It has been reported that strains belonging to O6, O15, and O75 serogroups possess specific virulence factors which confer them special invasive ability [23]. 56% percent of the* E. coli* isolates did not type with above used sera, suggesting that large proportion of UTIs in the community is caused by other serotypes also. This underscores the need for further studies to establish the origins and transmission of these strains. The antimicrobial resistance in typable strains was found to be similar to nontypable strains showing high level of resistance to cefotaxime, norfloxacin, nalidixic acid, cotrimoxazole, and amoxy-clavulanic acid and low level of resistance to nitrofurantoin (5.3%), piperacillin-tazobactam (13.3%), and meropenem (3%) among typable strains. Out of 33* E. coli* isolates which reacted with polyvalent serogroups, 19 (57.6%) were ESBL producers but relation between these serotypes and ESBL production was not found to be significant.

To conclude, judicious use of antibiotics according to hospital antibiotic policy should be done to prevent emergence of resistant organisms. Infection control measures should be properly implemented to prevent spread of ESBL and multidrug resistant strains in the hospital. However, only limited serotypes were tested in our study and further studies with large number of samples and complete serotyping of* E. coli* are needed.

## Figures and Tables

**Table 1:** Age and area wise distribution of *E. coli* among children (<10 yrs).

Age groups	Total	Male	Female	^*∗*^OPD	Ward	^*∗∗*^ICU
0-1 yrs	14 (18.7%)	10	4	4	8	2
1–5 yrs	29 (38.7%)	19	10	9	14	6
5–10 yrs	32 (42.7%)	12	20	12	19	1
*Total*	**75**	**41 (54.7%)**	**34 (45.3%)**	**25 (33.3%)**	**41 (54.7%)**	**9 (12%)**

^*∗*^OPD: out-patient department, ^*∗∗*^ICU: intensive care unit.

**Table 2:** Antimicrobial resistance profile among *E. coli* isolates.

*E. coli* (*n* = 75)	Resistance (%)	Typable strains (*n* = 33)	Nontypable strains (*n* = 42)
Ampicillin	73 (97.3%)	33 (100%)	40 (95.2%)
Gentamicin	35 (46.7%)	15 (45.4%)	20 (47.6%)
Amikacin	12 (16%)	6 (18.2%)	6 (14.3%)
Amoxicillin/clavulanate	72 (96%)	32 (97%)	40 (95.2%)
Trimethoprim-sulfamethoxazole	63 (84%)	26 (78.8%)	37 (88.1%)
Nitrofurantoin	4 (5.3%)	2 (6%)	2 (4.7%)
Nalidixic acid	74 (98.7%)	33 (100%)	41 (97.6%)
Cefotaxime	61 (81.3%)	30 (90.9%)	31 (73.8%)
Piperacillin/tazobactam	10 (13.3%)	5 (15.1%)	5 (11.9%)
Meropenem	1 (1.3%)	1 (3%)	0 (0%)
^*∗*^ *ESBL*	36 (48%)	19 (57.6%)	17 (40.5%)

^*∗*^ESBL: extended spectrum beta lactamase.

**Table 3:** Frequency of *E. coli* serogroups in children with urinary tract infection.

Serogroup	Number
O1	5 (15.1%)
O2	3 (9.1%)
O4	3 (9.1%)
O6	11 (33.3%)
O7	2 (6.1%)
O15	5 (15.1%)
O75	4 (12.1%)
Total typable	33 (44%)
Total nontypable	42 (56%)
